# Precise realtime current consumption measurement in IoT TestBed

**DOI:** 10.12688/openreseurope.15140.1

**Published:** 2023-02-06

**Authors:** Rihards Balass, Vladislavs Medvedevs, Andris Ivars Mackus, Juris Ormanis, Armands Ancans, Janis Judvaitis

**Affiliations:** 1 Cyber-Physical Systems laboratory, Institute of Electronics and Computer Science, Riga, LV-1006, Latvia

**Keywords:** IoT, TestBed, Current consumption, Realtime, Carbon Footprint, WSN, Measurement

## Abstract

**Background**: The Internet of Things, similar to wireless sensor networks, has been integrated into daily life of almost everyone. These wearable, stationary, or mobile devices are in multiple locations, collecting data or monitoring and executing certain tasks. Some can monitor environmental values and interact with the environment, while others are used for data collection, entertainment, or even life-saving. To achieve the wireless part of the system, the majority of sensor nodes are designed to be battery-powered. While battery power has become increasingly ubiquitous, it tends to increase the global carbon footprint of electronic devices. This issue can be mitigated by employing some form of energy harvesting so that batteries can be refilled and the gadget lasts longer, but this does not alter the reality that batteries are still used and eventually discarded.

**Methods**: In this paper, the authors emphasise the significance of power consumption in battery powered devices. To be able to monitor devices power consumption, one of the measurable parameters is current. When users know the exact current consumption, they can decrease it by polishing the program or tweaking the duty cycle, making radio transmit less data or less frequently, thus decreasing overall power draw.

**Results**: In order to simplify current consumption monitoring, the authors have developed a testbed facility that provides real-time current consumption measurements, which may be used to enhance the duty cycle and battery life of the afore- mentioned devices.

**Conclusions**: While minimising total current consumption is a great way to extend the battery life and, thus, the carbon footprint, the primary culprit in the Internet of Things is the radio communications. This transmission is the primary source of current consumption. By determining the exact amount of current drawn during transmission and adjusting it, users can significantly extend battery life.

## Plain language summary

When you look at everyday life, you can see that almost everyone uses or at least comes into contact with the Internet of Things (IoT) or wireless sensor network (WSN) technologies at some point. Seeing how fast these devices are becoming more popular and how many there are, the authors decided to bring attention to the problem of the carbon footprint these devices are creating. By introducing the second version of their testbed facility, the authors talk about how important it is to measure the amount of current used in real time for the Internet of Things and wireless sensor network devices during their development phase. When the developers know exactly how much current their product is using, they can improve their performance, make the batteries last longer, and in turn reduce their carbon footprint. In the article, the authors talk about the requirements for the new testbed facility and how they tested and chose the necessary components to find the ones that are needed.

## Introduction

The reduction of the electrical energy consumption to extend battery life of wireless devices is one of the main challenges for modern Internet of Things (IoT) systems, which are typically represented as a combination of a wireless sensor network (WSN) and a data processing block ^1, 2^. The total energy consumption of wireless IoT devices is affected by multiple complex factors: (i) the electrical design of the device ^3^, (ii) the efficiency of the firmware ^4^, (iii) duty cycle of sensor node activity ^5^, (iv) duty cycle of the wireless radio ^5^, (v) disturbances and interference from other devices deployed in the same environment ^6^, (vi) wireless packet collisions ^7^ and other, which are often noticed only after the deployment of IoT devices.

Because of the complexity of aforementioned factors the theoretical estimation of energy consumption in real environments is exceedingly complicated, but the empirical evaluation of these factors during the development phase is encumbered by the fact that IoT devices are frequently deployed in situations where they are not accessible and permanent connection to the power grid is not possible ^8, 9^, such as environmental or agricultural monitoring ^10– 12^. This can be solved by combining a system for electrical energy consumption monitoring and a wireless IoT testbed, that could be deployed in real environment for testing of different operating modes.

This paper describes the architecture and electrical design of a current consumption monitoring solution for the implementation in the "EDI TestBed", a testbed facility developed in the "EDI" - Institute of Electronics and Computer Science, Riga, Latvia, further referred to as TestBed V1. This work is build upon the previous iteration of testbed (TestBed V1), which consists of: (i) software controlled power management unit (PMU), (ii) digital voltmeter circuit, (iii) current consumption measurement system, and (iiii) full access remote control of the device under test (DUT).

Additionally, several lessons learned describing improvements and requirements for the next iteration of EDI TestBed, further referred as TestBed V2, is provided.

## Related work

Several current measurement systems suitable for IoT applications have been identified and described. First of all we will describe the testbed facility EDI TestBed developed by Institute of Electronics and Computer Science, in this article referred as TestBed, is extensively described in the available literature, general description is given by Ruskuls *et al.* ^13^, details about the current consumption measuring system are described by Lapsa *et al.* ^3^, furthermore Judvaitis *et al.* ^14^ provides the back-end system overview and their technical implementation, Salmins *et al.* ^15^ provides an overview of the mobility aspect and together with the future development plans and DevOps integration described by Judvaitis *et al.* ^16^ this forms the core of TestBed. Practical use cases and usage evaluation is provided by Judvaitis *et al.* ^17^, Elkenawy and Judvaitis ^18^.

TestBed V1 ^3, 13^ was good at energy consumption monitoring, but nowadays it has become obsolete, as limitations of range and accuracy are increasing with a power technologies evolution and expansion of IoT systems. TestBed energy consumption monitoring subsystems operates as follows:

- Configurable power supply can be managed to produce constant voltage in range from 0.78 *V* to 4.7 *V* by using adjustable Low-dropout (LDO) voltage regulator;
- Current consumption monitoring system range and accuracy depends on chosen ammeter circuit that acts as a double range shunt ammeter with the ability to choose the range by switching shunt value between 10Ω and 0.82Ω. The output voltage value is captured by 16-bit 500 *kHz* analog-to-digital converter (ADC) and interpreted as a current value by knowing shunt resistor value. Measured current range is between 0.1 *µA −* 100 *mA* with a maximum relative error of 0.4%

There are multiple versions of current measurement systems available for IoT systems. Although most of them lack one or more features that are available and ready to use in TestBed.

SPOT - scalable power observation tool ^19^, represents a low-cost (< 25$) device for testing low power IoT nodes. It measures current consumption using single shunt ammeter channel. Despite the cost, SPOT has an impressive sampling frequency value for 1 *MHz* and good current sense accuracy below 1 *µA* that makes it possible to accurately calculate overall energy consumption value. But SPOT still has two major disadvantages:

- It doesn’t have a built-in power management unit.
- Current measurement range is only up to 40mA that makes it useless for high current devices testing.

The Raspberry Pi compatible energy measurement platform for wireless IoT devices "EMPIOT" ^20^, just like TestBed, works using shunt ammeter approach. The main purpose of this platform is energy consumption testing for each node of an IoT system. The compatibility of EMPIOT with the Raspberry Pi platform allows it to design a wide range of experimental IoT systems. The shortcoming of this system accuracy - 0.1 *mA*.

SANDbed ^21, 22^ is the wireless sensor and actuator network (WSAN) development system which has a testbed approach with more use cases than regular current consumption logger. As with all testbed functions it has same issues caused by the compromises between price, functionality, and quality. This system has an unusual current range switching solution, it switches between three current ranges instead of common single or double range solution: 100 *mA*, 200 *mA*, 500 *mA*. But this solution also has a lot of limitations namely the limited current measurement accuracy, which equals to 2% and maximum sampling frequency value - 400 *kHz*;

RocketLogger ^23, 24^, is an open-source energy harvesting logger. Portability allows to use it for IoT system design. RocketLogger has significant advantage to all aforementioned solutions – it’s current measurement accuracy, it is obtained by using double current measurement range with a different ammeter technique for each channel.

- Shunt ammeter for a high current range (2 *mA −* 500 *mA*) with a shunt value 50 *m*Ω
- Feedback ammeter for a low current range (10 *nA −* 2 *mA*) with a feedback resistance 680Ω

For low current circuits, the measurement accuracy is 0.03% + 4 *nA*, and for high current circuit, it is 0.09% + 3 *µA*. Rocketlogger has a in-built configurable PMU with range from *−*5 *V* to +5 *V* and digital voltmeter with 5.5 *V* range and 0.02% + 13 *µV* accuracy that allows users to calculate consumed energy using voltage supply from both RocketLogger and external source.

Despite all of its advantages, Rocketlogger has too low a sampling frequency to calculate communication device energy consumption per bit, this limitation comes from 64 *kHz* ADC.

While TestBed V2 development previous experience and related works were taken into account to release non-compromised energy harvesting measurement system with a wide measurement range, high accuracy, and fast sampling frequency to allow wide IoT system type testing.

## Architecture

EDI TestBed has been in development for some time now ^13^, and throughout the years has been improved in a numerous ways ^3, 14– 18^. One of the key functions of TestBed has always been energy consumption monitoring, and this function has been improved in several iterations. While the base idea stays the same, there are several improvements to functionality by increasing the resolution and reliability of TestBed energy consumption measurement system.

**Figure 1.**
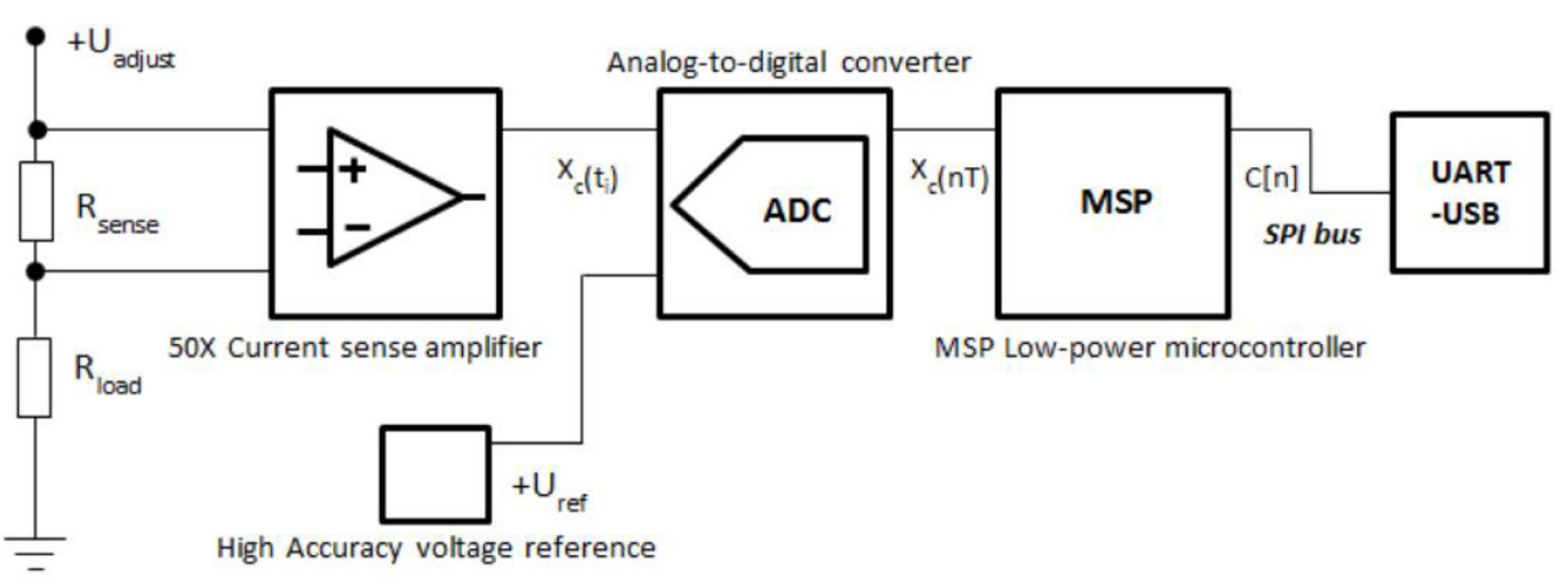
Basic block diagram of TestBed V1 current measurement circuit.

Figure 1 depicts the basic functionality of the TestBed V1 current measurement system. Current meter circuitry uses shunt ammeter approach ^3^ described in detail in Section.

While there are other available current measurement methods ^25, 26^, this was chosen due to its affordability and ease of use, as the number of components required to accomplish the desired outcome is relatively modest.

The TestBed V1 current measurement scale is set from 100 *nA* up to 100 *mA*. The goal was to measure the consumed current during the nodes duty cycle, which includes sleep and active mode. During the sleep phase, current consumption is measured in *µA* while during the active phase, which involves sensor data gathering and radio communications, current consumption can increase and is measured in *mA*. To be able to measure the current in a range so wide, there were two different precise resistors *R _sense_* placed in series with the device under test (DUT) ( *R _load_* ). The actual resistor used is selected based on the actual current consumed.

The desired power supply for the DUT is +5 *V* DC, which can be provided by an external power supply or a Universal Serial Bus (USB) 2.0 connection from a computer. To protect the computer’s USB controller, electrostatic discharge (ESD) protection diodes were fitted at the inputs of both power supply lines. The ESD protection diode changes the available voltage supply by reducing the source voltage by 0.5 *V*. According to the USB 2.0 specification ^27^, high-powered hub port voltage ranges from 4.75 *V* to 5.25 *V*, while low-powered hub port voltage ranges from 4.4 *V* to 5.25 *V*. Thus, the absolute worst-case scenario that is acceptable is if the hub’s voltage drops to 4.4 *V* , at which point, according to USB specification ^27^, only low-power functions can operate. Although reducing input voltage by 0.5 volts at input seems high, it is within acceptable range as it leaves 4.5 *V* to operate with.

The data sampling frequency in TestBed V1 is only 12.75 *kHz*. Due to the maximum reading frequency of 6,375 kHz according to the Nyquist theorem, this criterion is very imitating for use of the TestBed V1 for IoT applications.

$$f_{N}=\frac{f_{S}}{2}\;(1)$$

where

*f _N_* - Nyquist frequency that represents as a maximum reading frequency,

*f _S_* - bandwidth of the signal.

In this regard, the TestBed V2 maximum reading frequency increased to 500 *kHz* with 1 *MHz* sampling frequency.

**Figure 2.**
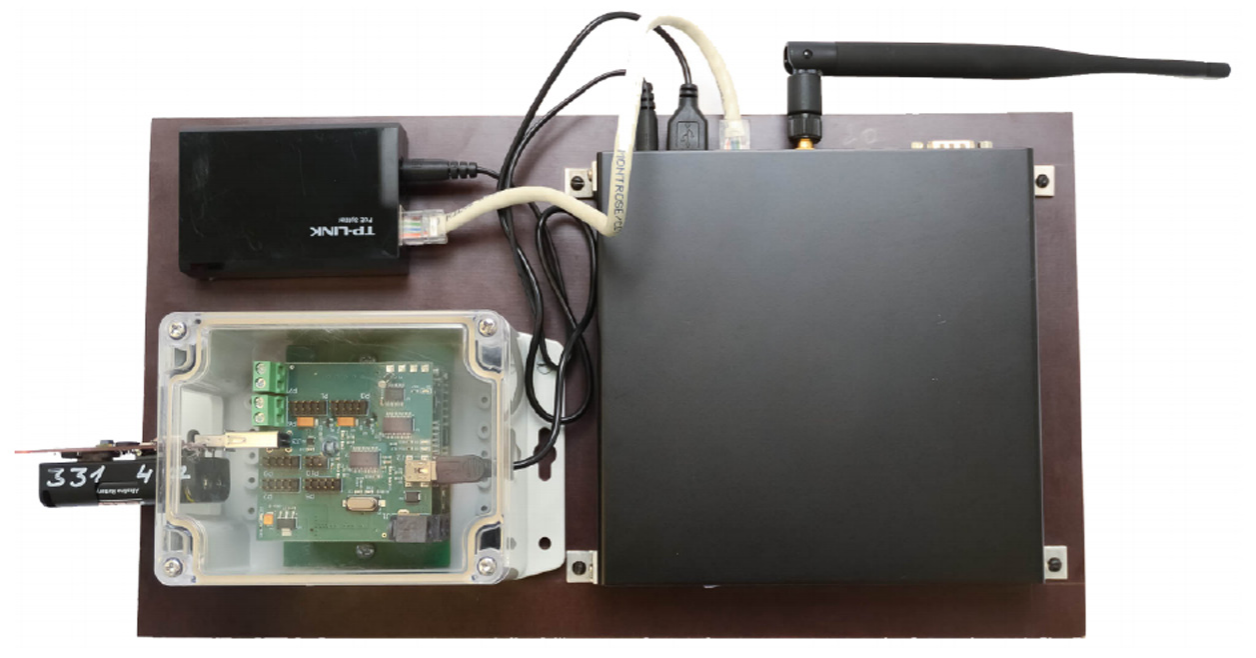
Stationary TestBed workstation.

**Figure 3.**
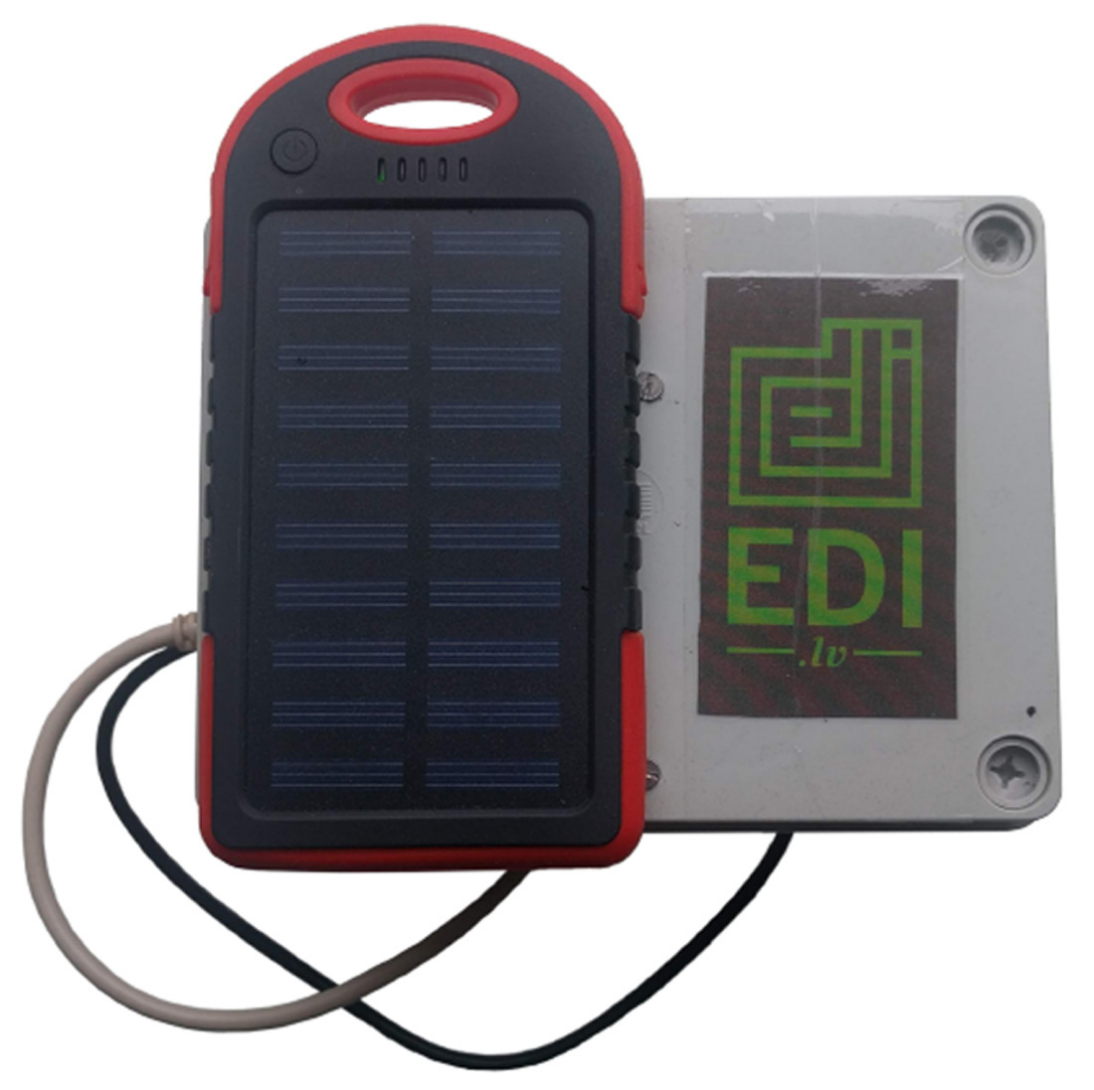
Mobile TestBed V1 workstation.

The TestBed V1 has two different variants: (i) Stationary ^13^, and (ii) mobile ^15^. A stationary testbed is placed throughout the EDI building and is supposed to be used only in laboratory conditions, see Figure 2. While the mobile testbed has Ingress Protection (IP) rating 54 enabled enclosure and is meant to be used outside see Figure 3.

### Practical lessons learned from original EDI TestBed

At the time of development, current consumption above 100 *mA* for a WSN node was considered too high, but recently it has become more common to get devices with current consumption much higher than 100 *mA*. For this reason, the 100 *mA* range is defined as a shortcoming and the authors aim to increase it in the latest revision. In addition, the lowest readable current has a 100 *nA* value, this value also has to be improved. For example, the STM32L476xx Microcontroller (MCU) in ultra low power mode can consume as little as 30 *nA* of current ^28^.

ESD protection diodes protect the power supply but does reduce the input by around 0.5 *V*. While, according to USB 2.0 specification ^27^, this is within the acceptable range, it makes the system provide power only to low-power functions. While utilising TestBed V1, we observed that in some instances, the DUT did not function properly, rebooting at, what appeared to be, random intervals or failing to turn on at all. Later, we discovered that there were extra 200 – 400 *mV* voltage drops along the cables we used, which caused our DUTs to reboot when more power was required or not power up at all when the voltage losses were bigger. In later stages of development, we were able to replace our cables and improve power reliability ^3^.

The original current measurement system is suppressed by the small voltage spectrum. While it works on all USB 2.0 devices, it does exclude some USB 3.x devices and different automotive and urban devices. At the time of creation, 5 *V* systems were chosen due to the USB standard, but a closer study reveals that the power supply range should be changed, widening the available spectrum from 3.3 *V* to 15 *V* to expand support for additional devices, as common IoT hardware has similar power requirements ^29^. In addition Power over Ethernet (PoE) has been increasingly used in home security and has been used in other fields ^30^.

### Requirements

This section describes the set requirements for the new TestBed V2, taking into account the latest research and lessons learned from TestBed V1.

### Functional requirements:

Functional requirements for the new TestBed V2 describes basic functionality of what the system is supposed to do and its parameters. While we do describe the whole system, the main premise is the current consumption measurement system.

***PMU requirements:***

- The TestBed V2 adapter provides the DUT with a controlled voltage source;
  This indicates that the voltage input to the DUT is precise and stable.
- The TestBed V2 adapter can simulate battery charging and discharging;
  This requirement remains the same as in the TestBed V1, so that the user may see what to expect from a device as the battery voltage drops by simulating battery discharge.
- The TestBed V2 adapter can be powered by a variety of external power sources;
  If the TestBed V2 adapter can only be supplied by grid power, this can be troublesome, hence additional power supply choices, such as PoE, battery, and external power adapter, must be available.
- To power the TestBed V2 adapter multiple power sources can be used simultaneously;
  This need is precautionary, so that when the TestBed V2 adapter is simultaneously attached to battery and external power supply, there are no conflicts.
- The TestBed V2 adapter can measure the DUT current consumption in real time.

***Connectivity requirements.*** Certain criteria have been established to improve TestBed V2 connectivity with DUT. While using TestBed V1, we realized that various wired and wireless connection protocols would be desirable in addition to the USB serial communication option.

- The TestBed V2 adapter uses standardised wired and wireless digital interfaces to communicate with the DUT and the testbed server;
  To reduce the likelihood of user error, communication must be established in accordance with specific standards, such as supplying the USB 2.0 Type A connector with only 5 *V DC*. In addition, wireless connectivity is being added between the TestBed V2 adapter and the DUT. All employed communication protocols and interfaces must adhere to predetermined standards.
- The device can be used as a standalone device.
  The TestBed V2 adapter consists of numerous modules, such as the power supply and current measuring module. Each of them should be capable of functioning independently. The user should be able to utilize the current measurement module without the TestBed V2 power source, instead powering it directly from a personal computer or laboratory desktop power supply.

***Debugging interface requirements.*** Requirements for DUT debugging during development life cycle. There are numerous debugging methods, and their respective requirements for successful and straightforward debugging are outlined here.

- Debug and update DUT firmware remotely;
  Important characteristics of testbeds include the capacity to remotely update and reprogram the DUT. This useful feature permits the simultaneous reprogramming of many DUTs.
- Reset the DUT operation remotely;
  This requirement is useful if the DUT has crashed and is unresponsive, in which case a physical reset must be performed. In most circumstances, this is accomplished via a push button or switch on the DUT, but in our scenario, the reset must be toggled remotely.
- Serial communication channel between the DUT and a remote client/device;
  A DUT has to appear as a serial communication device, as a seamless pass-trough of the chosen communication channel, this helps with development, as from the clients perspective the DUT is directly connected to clients development platform.
- Logging capability for the DUT.
  A complete log file of data input and output from DUT.

***Casing requirements.*** The TestBed V2 adapter has safety mechanisms for protecting its hardware and connected DUT’s in dangerous operational conditions.

IP 54 is the most common rating for devices and is the easiest to achieve, the original TestBed already achieved this IP rating. But it only protects the device from dust and splashing water ^31^. If the TestBed V2 should be used in a more moist environment, for example a swamp, a lakeside or in a strong rain, than the rating must be up to IP 57. The protection from dust stays the same, but in addition to that we are increasing protection from being submerged in water up to 1 *m* in depth. In addition, dangerous operational conditions also includes protection from overheating.

### Operational requirements

The requirements on how and in what conditions the system is supposed to operate is described further. From theoretical point of view the requirements related to the current consumption measurement of Internet of Things devices have increased recently, the most demanding hardware can achieve as low as 0.03 *µA* current consumption in most aggressive current saving mode ^32^. On the other hand, the narrow Band Internet of Things devices can consume up to 280 *mA* ^33^. The voltage of some of the mentioned Internet of Things devices can go from 3.3 *V* up to as high as 15 *V* ^34^. Based on the latest IoT devices available on the market we have set a demanding **list of requirements for the PMU**:

- Output current range up to 1 *A*
- Output voltage range 0 *V* - 34 *V*
- Current measurement range 2 *nA* - 3 *A*
- Current measurement frequency 1 *MHz*
- Current measurement accuracy 100 *pA*

### Power supply unit architecture

The TestBed V2 is intended to run from a number of power sources, including POE up to 100 *W*, USB type-C with power delivery support up to 100 *W*, and a barrel jack connector: voltage range of 4.5 *V* to 36 *V*

TestBed V2 can also be utilised as a power supply for connected DUT *via* TestBed Adapter. Thus, TestBed V2 PSU is a universal power supply with an intuitive interface, as it accepts a range of input power sources and prepares them for DUT.

As the TestBed is designed to represent a wide range of operational conditions, authors have distributed the TestBed adapters throughout the EDI main building, including outdoors. TestBed V2 offers control over output voltage and output current, as well as the “stable mode” that attempts to adjust as much as possible for fluctuations in current and voltage. The output voltage and current of the power supply will depend on the power supply topology selected for the TestBed.

### High current measurement mode

**Figure 4.**
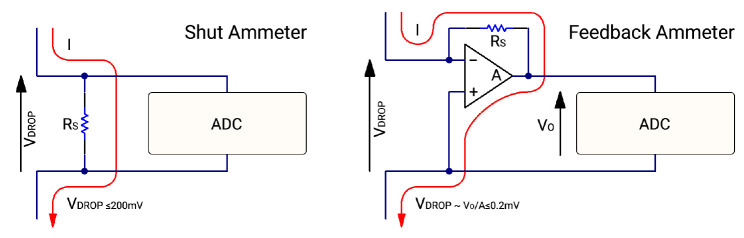
Shunt ammeter vs feedback ammeter.

For high current range measurements we are still using a shunt ammeter circuit see Figure 4. This method measures gained voltage across a shunt resistor. And the current ( *I*) measurement is obtained by dividing this voltage drop measurement ( *V* DROP) with the known value of the resistor ( *R*_S_).

$$I=V_{DROP}/R_{S}\;(2)$$

*R*_S_ is connected in series with a DUT that introduces a voltage drop error to the circuit. The weak point of this circuit can show up if *R*_S_ is too high compared with a load impedance. So shunt resistance should be as low as possible, to reduce voltage drop across it. For our implementation we have chosen a 50 *mΩ* resistor as a shunt resistor as it will create only 150 *mV* drop if there is 3 *A* load thru measuring circuit, this is specified upper limit of current measurement. Also 50 *mΩ* resistor provides capability to theoretically measure current form 1 *nA* up to 3 *A* using 32bit ADC if there is no noise but in real world its not possible due noise and voltage drift in whole measuring circuit. Calculations for this 50 *mΩ* resistor was done using spreadsheet for visually providing information about ranges, voltage drop and current capability, spreadsheet is in supplementary data ^35^ in the *Shunt-and-gain-calculations.xlsx* file.

### Low current measurement mode

In the low current architecture a feedback ammeter technique is used 4. The feedback ammeter’s most significant difference is smaller voltage drop ( *V*_DROP_), that makes it possible to measure smaller current changes with smaller error rate. The current ( *I*) measurement is obtained by dividing output voltage ( *V*_O_) with known shunt resistance ( *R*_S_)

$$I=V_{O}/R_{S}\;(3)$$

By utilising operational amplifier its also possible to get readings faster than using shunt ammeter measuring due to nature of voltage settling across resistor, if it is needed to measure small current by using shut ammeter method then shunt resistor ( *R*S) nominal must be high, and that with capacitance form wiring makes large settling time for such circuit.

## Simulation

During TestBed V2 development phase many main component simulations and evaluations were done, these simulations were performed to reduce prototyping iterations and to gain a deeper understanding of how the integrated circuit(IC) will perform in conjunction with other electrical components. The described simulations are published in supplementary data ^35^ in the *Simulations* folder. The circuit simulations for the most important parts of the developed adapter, with the respective file name in brackets, are as follows: (i) main power converter, TestBed V2 power supply part responsible for DUT power, multi power input rail part( *testbed_ideal_diode file*), "over the top" operational amplifier functionality( *opamp_adc_buf file*) and low current feedback amperemeter part( *feedback_amp file*).

TestBed V2’s main power part was simulated due need of checking for noise, “Voltage for Input-to-Output Control” and how positive and negative power rails will work. This consisted of buck-boost converter LT8210 ^36^ *testbed_main_bb_positive_negative file* which is used to adjust voltage accordingly from one of used input rails. Power is regulated accordingly to next power paths component - LDO LT3045-1 ^37^, this is achieved by utilising special LT3045-1 functionality “Voltage for Input-to-Output Control” (VIOC) witch controls converter before LDO so that LDO doesn’t need to deal with huge voltage difference between input and output of it as LDO by its principle converts voltage difference in heat, furthermore this reduced whole system power and heat waste. The LDO choice was also based on noise parameter as chosen LDO also has very low noise in output. As this part is also responsive for negative power rail there is another voltage converter ^38^ witch is in inverter mode to produce negative voltage from positive rail. On the negative voltage circuit buck-boost converter was also used together with negative voltage LDO ^39^ using the VIOC feature, also to reduce heat and power waste in system. Resulting circuit produces positive and negative voltage rails that further is used in measurement circuits as they provide very low noise due design and component choices.

During development and simulation process for DUT power supply *testbed_psu_filter file* a simple approach was chosen, using LT3081 ^40^ which can be regulated from Digital to Analogue converter(DAC). DAC wasn’t included is simulation as simulation as it wasn’t necessary. Also LDO should have the potential to regulate the voltage from 0 to input rail voltage. In simulation regarding this part protection against scenario where DAC could provide higher voltage than power supply voltage was included using Over the top operational amplifier LT6015 ^41^. In case if DUT should have very stable voltage output there is implemented capacitor filter for power rail fluctuation if needed. For the TestBed V2 input power rails there was a need to check and simulate if system could be powered from different power paths simultaneously not damaging device itself and also not damaging the power rails. Such option was simulated with so called ideal diode LTC4359 ^42^ which instead of real diodes uses mosfets to turn on or off power rails according to input rail state. For testing purposes a simulation was created where operational amplifier LT6015 characteristics were tested together with the earlier mentioned over the top feature. Another simulation was created for low current measurement circuit with ultra low noise operational amplifier ADA4522 ^43^ whose output was fed in ADC driver LTC6363 ^44^.

## Evaluation and results

In this section authors describe how they developed the TestBed V2 according to requirements and lessons learned from TestBed V1. A brief introduction to the system as a whole is given, with the main emphasis on current measurement system.

The important concept that was kept in mind during the development of the TestBed V2 was versatility and as such diverse supply voltages and operation conditions were the primary features that were maximised.

TestBed V1 utilises a "multi-processor for multi-function" approach ^13^, which results in an exponential increase in complexity with each new feature. In contrast, the new TestBed V2 architecture is planned to utilise a single microprocessor that is powerful enough to perform data processing and support all required TestBed V2 features, increasing the data processing speed and streamlining the system development. To develop TestBed V2 current measurement system we took inspiration from the RocketLogger architecture Described in Section, which enables measurements in two ranges: (i) ultra-low current consumption measurement for sleep mode performance study and (ii) normal operating mode for active state analysis. This approach gives the ability to seamlessly switch between two modes, which gives uninterrupted data to user about the DUT’s current consumption. This means that there is no need to manually switch the current measurement mode while DUT is in sleep or active mode. The two different current measurement modes utilise two different current measurement techniques: Shunt ammeter for a high current range, and feedback ammeter for a low current range.

The TestBed V2 adapter inherits the same paradigms as the TestBed V1 itself. The adapter’s primary objective is versatility and with this in mind, the primary focus has to be on developing user-friendly software for TestBed V2 adapter, which works as a bridge between the TestBed V2 infrastructure and the DUT. The DUT can be operated *via* the TestBed V2 adapter either as a USB device or as an independent device linked *via* a terminal block. If the USB topology is employed, it is possible to connect with the DUT *via* the Testbed V2 adapter using the built-in USB hub or to move the DUT to an independent USB port using pass-thru mode, where another device communicates with the DUT.

### Hardware choices

In the initial phase of development we reduced the number of available ADC’s and MCU’s that are compatible with our requirements Described in Section by looking at their theoretical characteristics. Later we built a breakout boards with chosen ADC’s and operational/instrumental amplifier to test real world performance, to see if by some means the said theoretical performance, like sample frequency in conjunction with test MCU’s and single board computers (SBC) *e.g,* Raspberry PI, RockPI, could not be achieved.

ADC for current measurement system was chosen to fulfil predefined requirements but also considering MCU capabilities to be reasonable. As potential candidates the LTC2335-18 ^45^ and LTC2500-32 ^46^ were chosen. LTC2335-18 was considered due 1 *MHz* data acquisition speed for measurements, 8 channel multiplex for flexibility and possible design simplicity, 18bit sampling resolution and SoftSpan feature. SoftSpan enables each output range to be configured via software individually. This can also reduce the complexity of the Printed Circuit Board (PCB). LTC2500-32 on the other hand was also chosen due 1 *MHz* data acquisition speed and 32bit precision filtered output capabilities. Both ADC supported 100 *MHz* SPI data transfer frequency that could be sufficient to manage data transfer frequency of 1 *MHz*.

**Table 1.** ADC parameters.

Parameter	LTC2335-18	LTC2500-32
Data transfer frequency	1MHz	1MHz
Channels	8	1
Resolution	18	32
Data transfer frequency	100 MHz	100 MHz

As the TestBed V1 was multi-processor system, we decided to redesign everything to be more centred but with more powerful microprocessor. The options were narrowed down to single board computers that could run Linux distribution and had good documentation, (i) Raspberry Pi 4 ^47^, (ii) Hardkernel Odroid C4 ^48^, (iii) Radxa RockPi 4 ^49^ and

Nvidia Jetson Nano ^50^. At the end Nvidia Jetson Nano was chosen due to the included Compute Unified Device Architecture (CUDA) cores, that offer possibilities to develop machine learning algorithms and systems powered by artificial intelligence.

### Printed circuit board

**Figure 5.**
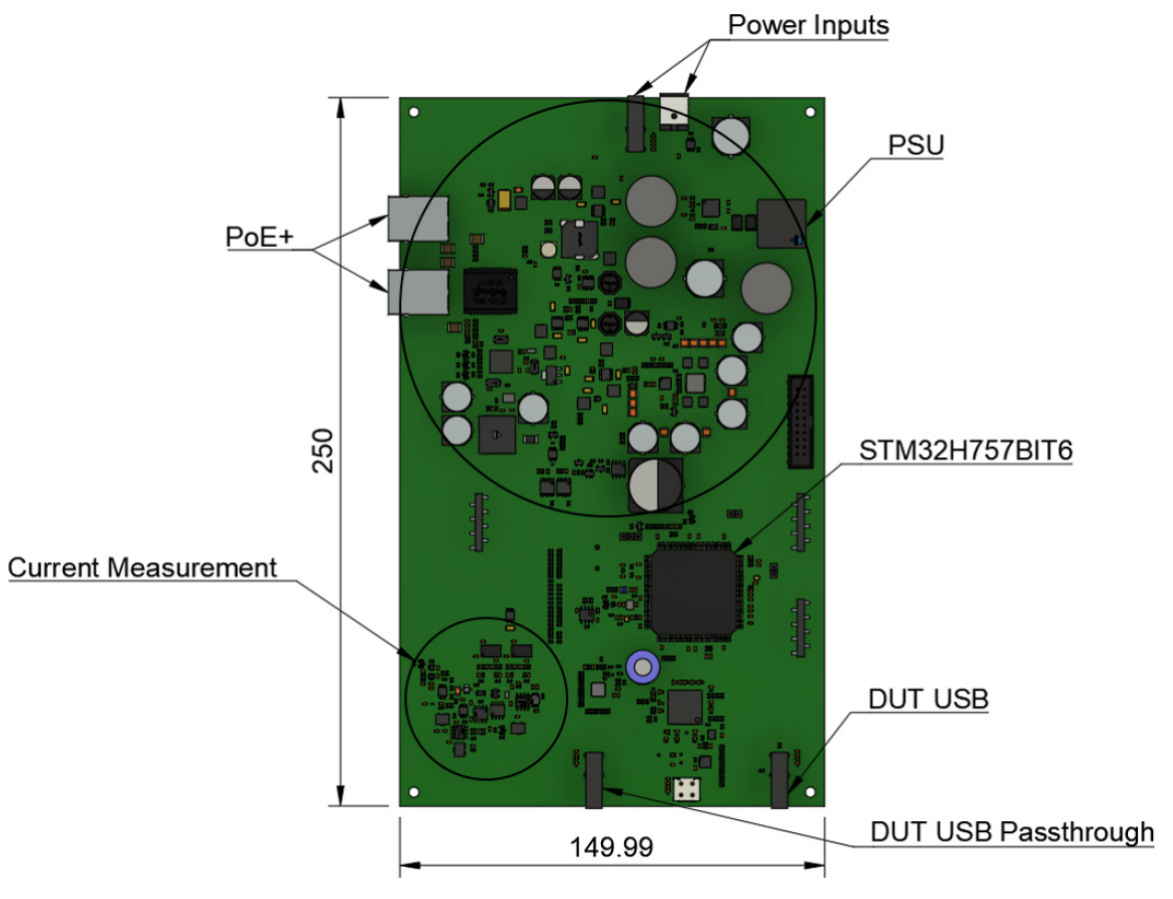
TestBed V2 Printed Circuit Board 3D design.

From acquired requirements authors designed a six layer PCB with "Altium Designer" (industry standard for PCB Design) ^51^, with open sorce alternatives awailable, like "KiCad" ^52^, including power supply unit as-well as current measurement system as a single board unit. The necessity to migrate from two layer PCB of TestBed V1 to six layers on TestBed V2, was the increased complexity of new PCB. This PCB is designed to interface with Jetson Nano and act as a bridge between both DUT and Jetson Nano. In Figure 5 is seen the 3D design of said PCB designed with "Autodesk Fusion 360" ^53^ software, with open source alternatives available, like "Blender" ^54^ or "FreeCad" ^55^.

### Casing

**Figure 6.**
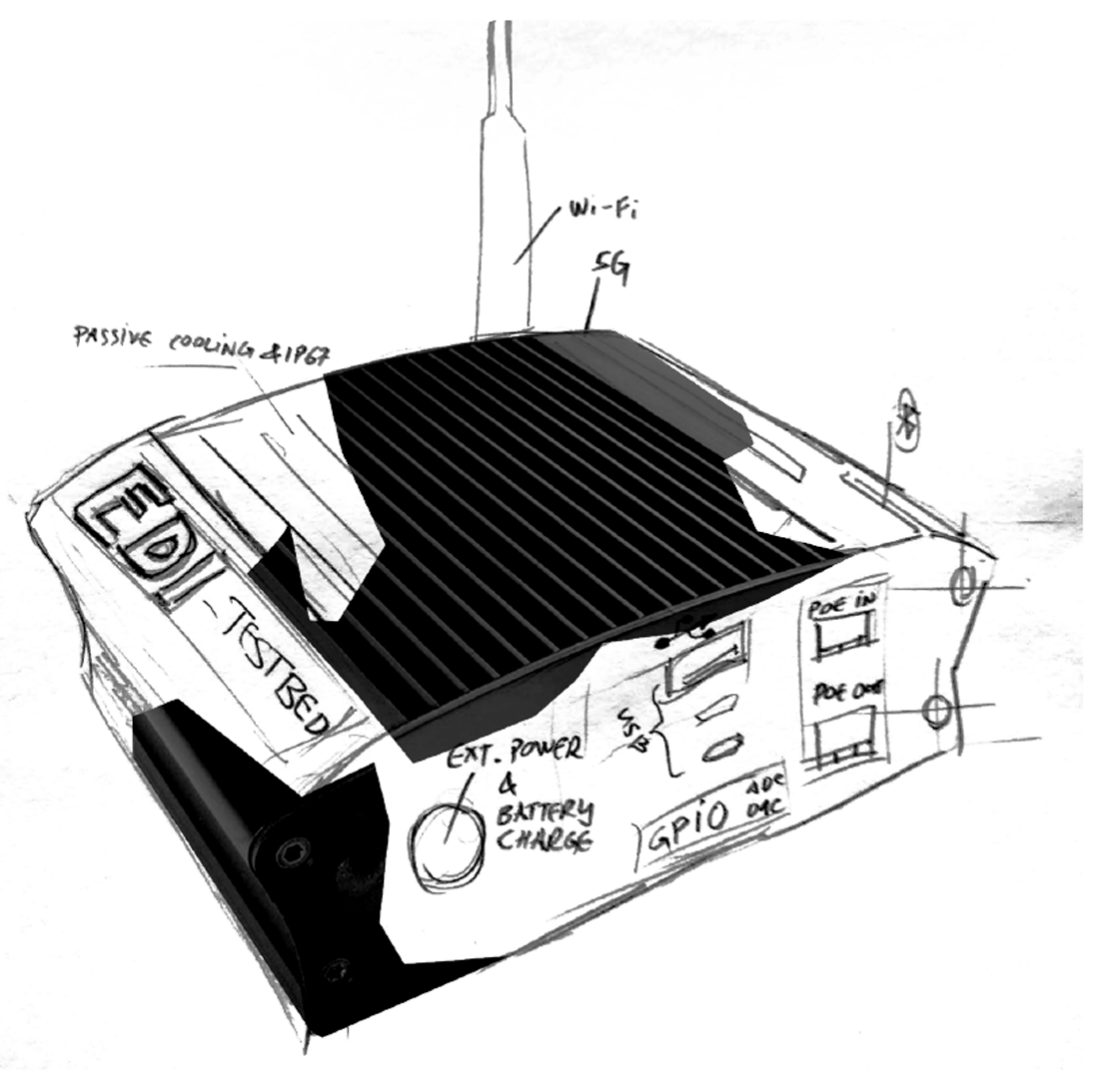
"TestBed V2" visual sketch.

First sketch of TestBed V2 design was made on a piece of paper see Figure 6. In the design there can bee seen that the enclosure is supposed to act as a passive cooling system for the whole device, therefore improving the thermals and IP rating, because there are no holes for air cooling. All of the necessary ports are routed to the side and are meant to be sealed. To improve the radio coverage the antennas are pulled out of the enclosure, so that no metal enclosure would harm the radio reception.

**Figure 7.**
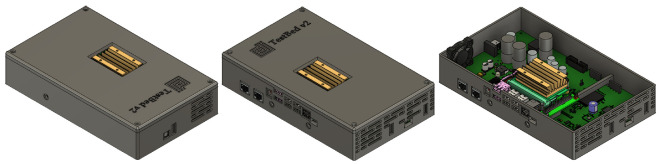
"TestBed v2" prototype 3D designed enclosure.

The next step was to use 3D computer-aided design (CAD) software to design a suitable enclosure for TestBed V2, taking into account the PCB that had been designed, see Figure 5, and the fact that the first prototype will be 3D printed using ABS Plastic. There are only TestBed V2 components included inside the enclosure at this stage power supply, current consumption monitoring system and central processing unit, which is the Nvidia Jetson Nano ^50^ developer kit, see Figure 7. As in the sketch all of connectors are pulled to the side of enclosure. As this enclosure is supposed to be plastic, it is impossible to cool the system using it as a housing, so one side of the enclosure has been designed so that air could flow freely cooling the Jetson Nano and other components on the board. To increase the airflow additional cooling fan is attached and fitted to one of the sides see Figure 7.

To test both ADC’s 1 in question authors set up a test bench where both ADC’s were mounted on breakout boards with additional components as per both ADC’s datasheets and wiring underneath for communication and data acquisition. 8 During testing of 32bit ADC there were multiple hardware and software iterations during selecting adequate MCU and software combination, the software used in the tests is added as additional data to this publication ^35^. The first tests were performed on Arduino DUO platform using SAM3X8E 32bit ARM Cortex M3 MCU. Those tests were for proof of concept to see if its possible to acquire any data and how does the ADC perform. During testing ADC performance and communication we also used DSLogic U3Pro16 logic analyser MCU where the clock source and logic analyser was used as the data acquisition device. Only proof of concept data was captured as screenshots during XM1000 mote bootup stage via low speed USB connection. 9

**Figure 8.**
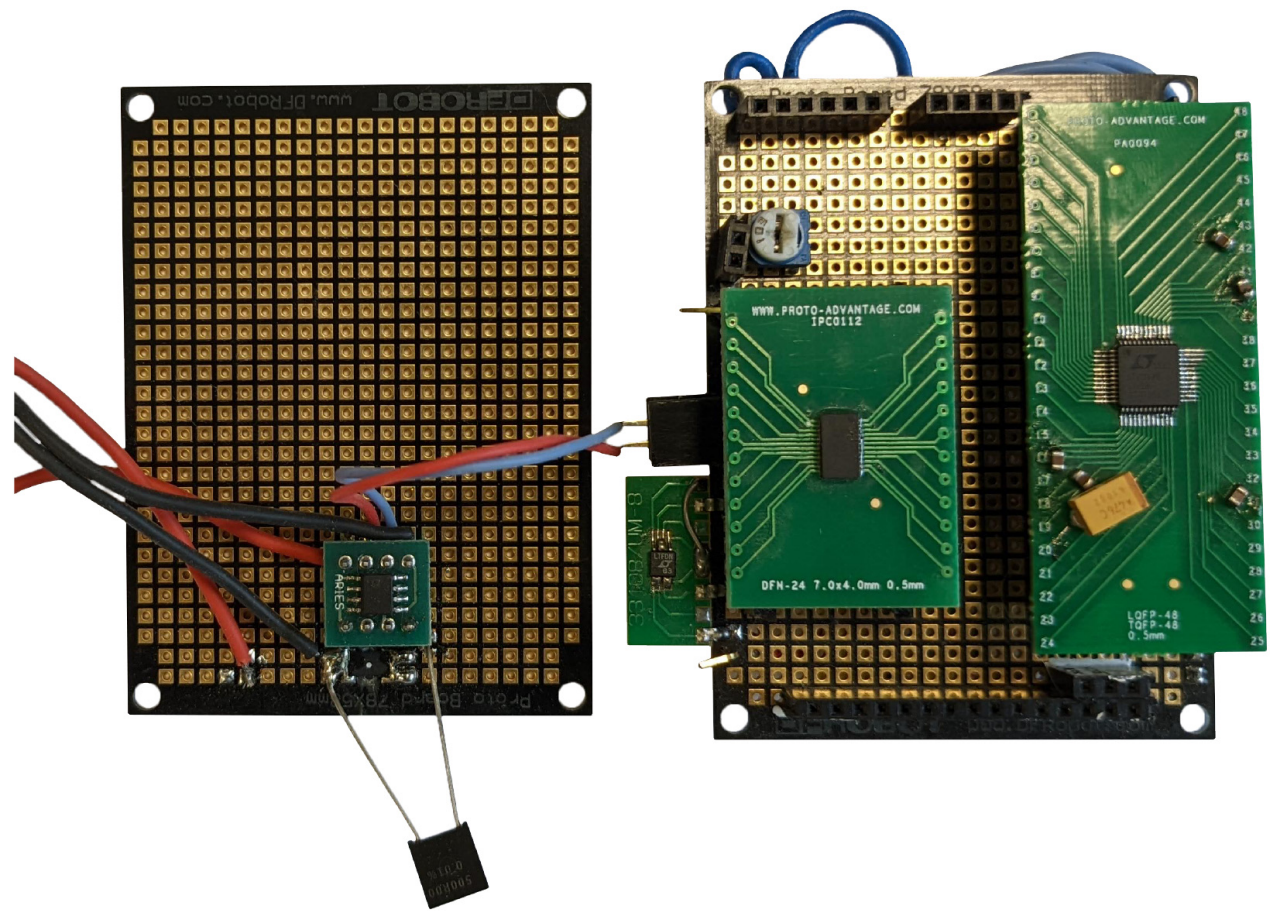
Testing breakout board.

**Figure 9.**
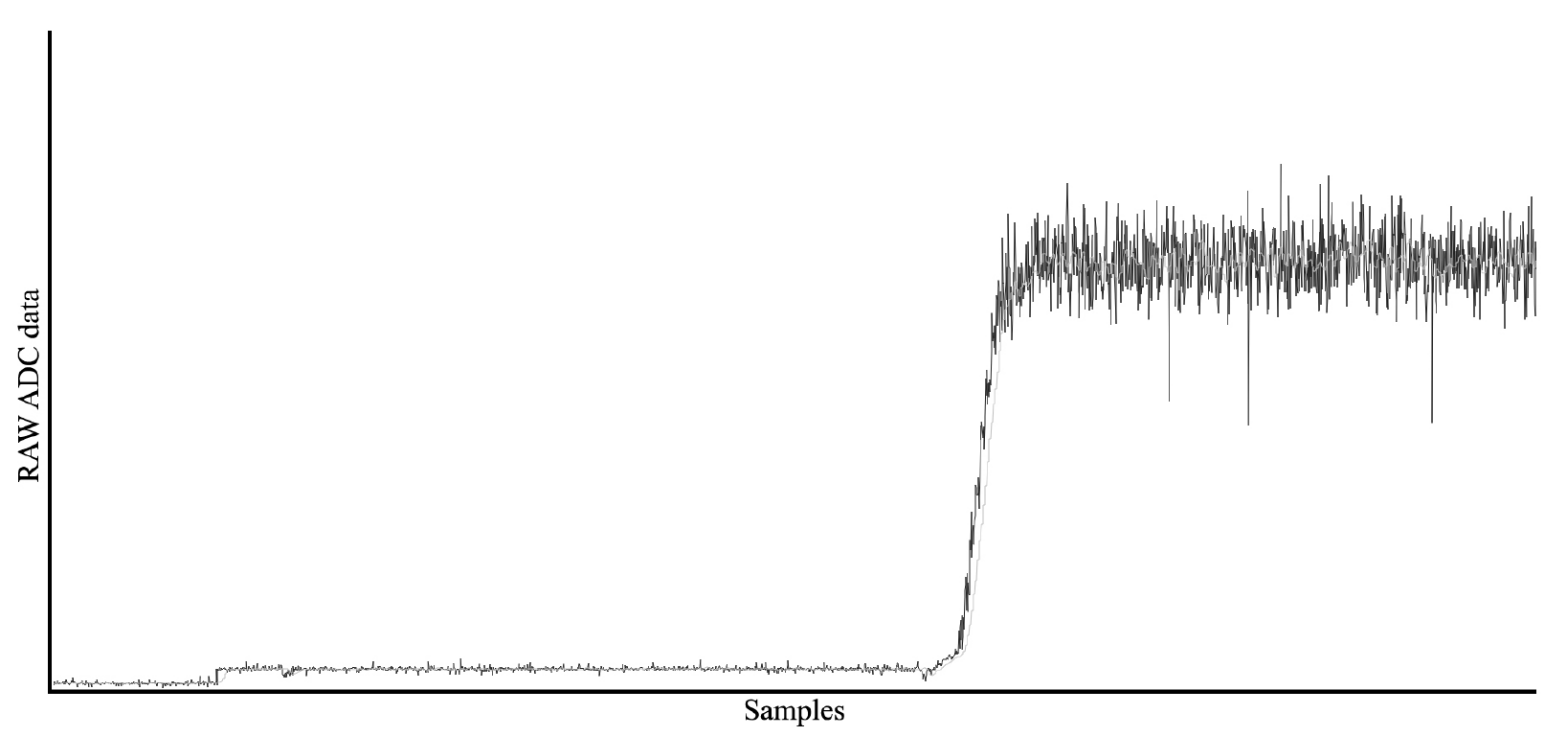
Captured proof of concept data ^35^.

During testing and while choosing the final platform for ADC communication, there also were tests on RockPI and Raspberry PI platforms. Authors tested maximum data acquisition speed from ADC using SPI protocol and hardware connection between ADC and test platform. Additionally to SPI protocol hardware pinout there were used ADC specific trigger pins as BUSY, CLOCK, DRL to be aware of ADC state. State of ADC was important as as during ADC sampling there should not be data reading as it renders ADC sample invalid. BUSY signal meant that ADC is in sampling phase, CLOCK signal additional to SPI clock signal was used as ADC sampling start signal and DRL signal was used as flag to determinate available data on ADC for reading. From platform side of tests this was done by utilising kernel module functionality to test platform capability of driving ADC and receiving data streams, used test code is published in supplementary data ^35^ in the *Testbed V1 SBC modules* folder. Unfortunately it was not possible to reliably utilise them even in real time kernel, as the maximum possible frequency of data would be only *∼* 300 kHz on ADC not considering actual data reading. ADC sampling takes around *∼* 600 *ns*, while less than 400 *ns* are required to establish the SPI data connection, retrieve data, and prepare for the next transfer. If data is read during an ADC sample cycle, it becomes invalid due to SPI transmission and mixed data stored in the memory buffer of the ADC. In addition, at these sample frequencies and with the SBC continuously triggering for data read, since the ADC triggers an interrupt signal on the SBC for data transfer, the SBC became unusable and unresponsive for any other job. During these tests, the authors decided to discard the concept of using any SBC to communicate directly with ADC from the MCU.

During researching of other available data reading options from ADC authors tested different MCU and SBC options like Arduino DUE core board.

Using this "Arduino DUE core" board in conjunction with ADC breakout board 8 by utilising same data acquisition and ADC triggering methods as earlier mentioned with SBC’s except for kernel module part as its not applicable to test MCU it was possible to acquire data with a sample frequency of 300 *kHz* not only triggering as it was with SBC’s. Additionally it was also possible to explore each ADC features such as data averaging and filtering in case of 32 bit ADC by utilising second SPI data channel of ADC, all of features is available in ADC datasheet ^46^ and commented on test code that is published in supplementary data ^35^ in the *Testbed V1 due core test* folder. However, given the output capabilities of chosen ADC and requirements described in Section , this sample frequency was insufficient. The MCU board based on an STM32H743VIT6 was the next board evaluated, used test code is published in supplementary data ^35^ in the *STM32H743 CubeMX test* folder. Additionally to same testing scheme as it was done with "Arduino DUE core" board there was used option to ignore data ready flags, but instead generate clock for triggering ADC sampling and rely on manufacture defined timing constrains of ADC, so that after triggering ADC sample wait in idle at least 630ns and then without reading any data ready flag assume that data has been captured and use DMA to offload Central Processing Unit (CPU) with direct registry writing and reading to further offload CPU processing cycles, the authors were able to achieve with this schheme a sample frequency above 900 *kHz*. Research revealed that this was one of the most powerful MCU’s on the market at the time in terms of MCU clock speed, pin count, and implementation, so the decision was made to stick with this type of MCU that was a little bit more powerful so that it could perform not only data acquisition but also other tasks, like DUT power supply management and data processing for host system over USB. MCU developed by STMicroelectronics were used after exhaustive research into quicker and more accessible computer platforms. As its clock frequency was 480 *MHz*, GPIO frequency was also significantly increased.

## Discussion

Even though the EDI TestBed V2 is not quite complete, we were able to collect sufficient data ^35^ to verify that we are moving in the correct direction. In future iterations, we intend to isolate the current measurement device and the power supply unit onto different PCBs. When designing both of them, we noticed that the majority of components and functionality required for the power supply unit are already populated on the current consumption PCB, therefore the decision was made to integrate them onto a single PCB for prototyping and usability considerations.

The EDI TestBed v2 could be utilised to enhance current wireless networks as well as for the creation of new ones. Our current consumption measuring system can identify the highest consumers and time periods, allowing users to utilise the data as they see fit. For instance, one could determine that it is preferable to use an additional ULP MCU in addition to the main MCU, so that the main MCU can be shut down for the duration of sleep, or that there is no need, since the MCU used in the system has a superior sleep mode than the ULP MCU, and waking the MCU can actually decrease the battery’s lifetime. Due to the variety of use cases, these findings may vary, for instance, there may be varying needs for the frequency of sensor readings, resulting in variations in duty cycle.

## Ethics and consent

Ethical approval and consent were not required.

## Data Availability

Zenodo: Supplementary data for Precise realtime current consumption measurement in IoT TestBed publication. https://doi.org/10.5281/zenodo.7417349 by Balass *et al.* ^35^ This project contains the following extended data: Simulations (folder containing simulations of circuits of TestBed V2) STM32H743 CubeMX test (folder containing code) Testbed due core test (folder containing code) Testbed SBC modules (folder containing code) Shunt-and-gain-calculations.xlsx (Data for gain and size of shunt resistor) Data are available under the terms of the Creative Commons Zero "No rights reserved" data waiver (CC0 1.0 Public domain dedication).
