# Supplementary material for: Precise realtime current consumption measurement in IoT TestBed
Source: Open Res Eur. 2024 Jan 25;3:27. Originally published 2023 Feb 6. [Version 2] doi: 10.12688/openreseurope.15140.2 (PMC11069045; doi:10.12688/openreseurope.15140.2)
Supplement: Peer review history

# Reviewer report: Reviewer response for version 2

**DOI:** 10.21956/openreseurope.18321.r38664

**Published:** 2024-05-03

**Authors:** Mun Hou Kit

**Affiliations:** 1 Department of Mechatronic and Biomedical Engineering, Universiti Tunku Abdul Rahman, Petaling Jaya, Selangor, Malaysia

**Major comments:**

 The paper has compared the existing testbed facilities in current measurement capabilities. This paper also presented the lacking of the TestBed V1 and proposed a TestBed V2 with improved capabilities and additional functions. Some results have been presented such as the 3D design of the prototype and simulation and testing of the ADC and hardware component selection process. However, the development of TestBed V2 is not finished yet. This paper just showing that they are collecting sufficient data and moving in the correct direction. The content is not reflecting the title of the paper “Precise realtime current consumption measurement in IoT TestBed” that should have the content of testing of the proposed IoT TestBed in realtime current consumption measurement.

**Minor comments:**

- “The block diagram was made to visualize the architecture of the design of TestBed V2 4.” Is 4 refers to Figure 4, if yes please rephrase it.
-  Please define “PW” in the basic block diagram ( Figure 4).
- The paper mentioned that the TestBed V2 adapter can simulate battery charging and discharging but no result of simulation is shown in the result and discussion section.  
- Numerous typo errors in this paper. Some examples as below:

“The TestBed V2 adapter inherits the same paradigms as the TestBed V1 itself 3.2.” what is the 3.2 in this sentence? “Described in Section 3.2 by looking at their theoretical characteristics.” No section 3.2 in this paper.

Is the study design appropriate and does the work have academic merit?

Partly

Is the work clearly and accurately presented and does it cite the current literature?

Yes

If applicable, is the statistical analysis and its interpretation appropriate?

Not applicable

Are all the source data underlying the results available to ensure full reproducibility?

Yes

Are the conclusions drawn adequately supported by the results?

Partly

Are sufficient details of methods and analysis provided to allow replication by others?

Yes

Reviewer Expertise:

Power monitoring and IoT

I confirm that I have read this submission and believe that I have an appropriate level of expertise to confirm that it is of an acceptable scientific standard, however I have significant reservations, as outlined above.

# Reviewer report: Reviewer response for version 1

**DOI:** 10.21956/openreseurope.16378.r32548

**Published:** 2023-07-24

**Authors:** Marco Eugeni

**Affiliations:** 1 Universita degli Studi di Roma La Sapienza, Rome, Lazio, Italy

IoT devices which are deployed in real-environment and where there is no access to the power grid need to have their power consumption measured for evaluation and design purposes. A theoretical estimation of the current consumption in real environments is a very complex process and for this reason the authors propose the use of a real-time testbed system which can help to provide an estimate of the current usage based on the real environmental conditions.

 The authors have described TestBed V2, which is an improved version of Testbed V1. The reviewers acknowledge that a lot of efforts was put in place for the design of the system, however the paper seems incomplete as it lacks a clear mention of performance results (graphs/tables) that can be compared to state-of-the-art systems.

 Major revisions should be applied to the paper.

**Major comments:**

- The literature review needs to be more extended to include more recent papers. Some references go back to 2009 (SANDbed [21][22]). For example the following paper could be assessed by the authors: Y. H. Tehrani and S. M. Atarodi, "Design & Implementation of High Dynamic Range Current Measurement System for IoT Applications," in *IEEE Transactions on Instrumentation and Measurement*, vol. 71, pp. 1-9, 2022, Art no. 2003009, doi: 10.1109/TIM.2022.3169538 ^1^.
- In the paper it should clearly be indicated what is the novelty / the differences between the presented system and the state-of-the-art solutions.
- The paper should also include a table / graph comparing the performances of the proposed system with the ones available in the literature.
- A system architecture diagram of the testbed V2 should be included to add more clarity on the system functions and input/outputs, etc.
- The discussion section should provide an analysis of the results. 
- A conclusion section is missing.

**Minor comments:**

- In the introduction, the authors provide a definition of IoT in the following sentence: “for modern Internet of Things (IoT) systems, which are typically represented as a combination of a wireless sensor network (WSN) and a data processing block1,2”. Can you indicate where in these papers [1] and [2] such definition has been given?
- In the text, some references to sections miss the section title/number.
- Figure 2 appears in reference [13]. The figure caption should contain the reference where the figure was taken from.
- In Equation (1) Fs should be the sampling frequency.
- In some parts of the paper “Shunt ammeter” is written shut ammeter. Please correct. The English should be improved in some sections of the manuscript.
- Please check again in paper [19] the value of the Current measurement maximum range (It should be 45 mA).
- In Figure 9, the ranges and the units are missing in the axes.

Is the study design appropriate and does the work have academic merit?

Yes

Is the work clearly and accurately presented and does it cite the current literature?

Partly

If applicable, is the statistical analysis and its interpretation appropriate?

Not applicable

Are all the source data underlying the results available to ensure full reproducibility?

Yes

Are the conclusions drawn adequately supported by the results?

Yes

Are sufficient details of methods and analysis provided to allow replication by others?

Partly

Reviewer Expertise:

Smart Manufacturing for space systems, IoT, space structures

I confirm that I have read this submission and believe that I have an appropriate level of expertise to confirm that it is of an acceptable scientific standard, however I have significant reservations, as outlined above.

## References

[1] : Design & Implementation of High Dynamic Range Current Measurement System for IoT Applications. *IEEE Transactions on Instrumentation and Measurement* .2022;71: 10.1109/TIM.2022.3169538 1-9 10.1109/TIM.2022.3169538

# Response

**Published:** 2023-12-05

**Authors:** Rihards Balass

Dear reviewer, thank you for the review! We have improved the manuscript as you suggested and considered your suggested improvements. We have also prepared the answers to the questions you have raised:

1. The literature review needs to be more extended to include more recent papers. For example the following paper could be assessed by the authors: Y. H. Tehrani and S. M. Atarodi, "Design & Implementation of High Dynamic Range Current Measurement System for IoT Applications"
  1. Thank you for suggesting the paper to look at, we have included it into this article, we have also been looking at more recent papers regarding the current consumption measurements in IoT systems. While there are multiple papers discussing the energy consumption of IoT devices, they are mostly about the current consumption for devices that are connected to the power grid.
2. In the paper it should clearly be indicated what is the novelty / the differences between the presented system and the state-of-the-art solutions.
  1. We have created a table that shows the differences between the identified testbed facilities with current measurement capabilities and the TestBed v1 facility.
3. The paper should also include a table / graph comparing the performances of the proposed system with the ones available in the literature.
  1. We have created a table that shows the differences between both versions of EDI TestBed. Together with the table described in previous comment, it can be seen how this research and the developed device impacts the IoT current consumption field.
4. A system architecture diagram of the testbed V2 should be included to add more clarity on the system functions and input/outputs, etc.
  1. We have added a block diagram of the proposed TestBed v2.
5. The discussion section should provide an analysis of the results.
  1. We have improved the discussion section with an analysis of the results.
6. A conclusion section is missing.
  1. We have added a conclusion section to the paper.
7. In the introduction, the authors provide a definition of IoT in the following sentence: “for modern Internet of Things (IoT) systems, which are typically represented as a combination of a wireless sensor network (WSN) and a data processing block1,2”. Can you indicate where in these papers \[1\] and \[2\] such definition has been given?
  1. There has been a mistake in the manuscript editing process which has been fixed. We have also rewritten the paragraph so it would be understood better.
8. In the text, some references to sections miss the section title/number.
  1. Since the Europe Open Research template does not contain numbering for the sections, this was missed. We have fixed the issue.
9. Figure 2 appears in reference \[13\]. The figure caption should contain the reference where the figure was taken from.
  1. The figure was made by one of the authors of this paper. Nonetheless we have added a reference to the original paper as well.
10. In Equation (1) Fs should be the sampling frequency.
  1. Fixed.
11. In some parts of the paper “Shunt ammeter” is written shut ammeter. Please correct. The English should be improved in some sections of the manuscript.
  1. We have gone through the paper and fixed spelling errors.
12. Please check again in paper \[19\] the value of the Current measurement maximum range (It should be 45 mA).
  1. Fixed.
13. In Figure 9, the ranges and the units are missing in the axes.
  1. We have added the missing axes, although they are not as clearly visible as we would like. To remedy that we added the explanation in the caption of the figure.

# Reviewer report: Reviewer response for version 1

**DOI:** 10.21956/openreseurope.16378.r32554

**Published:** 2023-06-27

**Authors:** Carmen Delgado Pinillos

**Affiliations:** 1 i2cat Foundation, Barcelona, Spain

This paper presents the design of a power meter for IoT devices. Since there are too many IoT devices, which are normally powered by batteries and these batteries have dangerous chemicals, being aware of the battery usage and how to make the best use of it is crucial. Authors try to solve this by proposing this device, capable of measure the current consumption.

 This is the second version of their testbed, and the reviewer has the feeling that the information is a bit mixed. The paper talks about V1 in order to explain the weaknesses that the V2 tries to solve, but this should be clearer explained. Authors could include a table with the differences of the two versions.

 The state of the art could be better formulated by making sure the differences between previous works are detailed. For example, when explaining 3, 13, 14, 15, 16 and 17.

 Apart from this, the details of the design and implementation are clearly explained and the device might be very useful in many applications.

Is the study design appropriate and does the work have academic merit?

Yes

Is the work clearly and accurately presented and does it cite the current literature?

Partly

If applicable, is the statistical analysis and its interpretation appropriate?

Not applicable

Are all the source data underlying the results available to ensure full reproducibility?

Yes

Are the conclusions drawn adequately supported by the results?

Yes

Are sufficient details of methods and analysis provided to allow replication by others?

Yes

Reviewer Expertise:

IoT, batteryless devices, resource allocation, energy modelling

I confirm that I have read this submission and believe that I have an appropriate level of expertise to confirm that it is of an acceptable scientific standard.

# Response

**Published:** 2023-12-05

**Authors:** Rihards Balass

Dear reviewer, thank you for the review! We have improved the manuscript as you suggested and considered your suggested improvements. We have also prepared the answers to the questions you have raised:  

1. The paper talks about V1 in order to explain the weaknesses that the V2 tries to solve, but this should be clearer explained. Authors could include a table with the differences of the two versions.
  1. We have created a table that shows the difference between both versions of TestBed.
2. The state of the art could be better formulated by making sure the differences between previous works are detailed. For example, when explaining 3, 13, 14, 15, 16 and 17.
  1. We have rewritten the text with emphasis on the differences and additions from each of the papers mentioned.
